# Case Report: Intermittent Chest Pain With Cough, Fever, and Pericardial Effusion Over the Course of 1 Year in an 11-Year-Old Girl

**DOI:** 10.3389/fped.2022.896824

**Published:** 2022-07-13

**Authors:** Jing Chen, Lu Qin, Lan-Fang Tang

**Affiliations:** Department of Pulmonology, The Children's Hospital of Zhejiang University School of Medicine, Hangzhou, China

**Keywords:** children, chest pain, pericardial effusion, paragonimiasis, misdiagnose

## Abstract

An 11-year-old girl presented with frequent chest pain, fever, and a cough that she had had for more than 13 months, as well as pleural effusion and large pericardial effusion. She was misdiagnosed with tuberculosis and received anti-tuberculosis drugs for 6 months. Within the past year, she also underwent two more thoracotomies and a thoracoscopic partial pericardiectomy. The final diagnosis of pulmonary paragonimiasis was established once it was known that she had eosinophilia, always drank stream water, and tested positive for antibodies against *Paragonimus*. Since antiparasitic praziquantel therapy was effective, paragonimiasis should be considered as a possibility in the differential diagnosis of tuberculosis in children.

## Introduction

Paragonimiasis is a zoonotic disease caused by lung flukes of the *Paragonimus* genus. Humans usually become infected by eating raw or undercooked freshwater crabs or crayfish containing the encysted metacercariae of these worms, as well as freshwater containing cysticercus (1). To date, more than 50 species of *Paragonimus* have been identified. The common species vary among different regions, where the most prevalent species in Asia is *Paragonimus westermani*, and the most prevalent species in Africa are *Poroderma africanum* and *Paragonimus uterobilateralis* (2). The parasite is endemic in South-East Asia, the Indian subcontinent, South and North America, and Africa. Meanwhile, paragonimiasis is prevalent in south-west China in the Sichuan basin (3). The clinical features of pulmonary paragonimiasis are non-specific and usually include a chronic cough, hemoptysis, pleurisy, chest pain, fever, and dyspnea. Chest radiograph and computed tomography (CT) images are non-specific and vary widely as well; they may present as patchy, cloudy, or linear infiltration of the lungs; pulmonary nodules and cavities; pleural effusion; and even masses (4, 5). These symptoms have often been confused with those of tuberculosis and lung carcinomas. Herein, we report on the case of an 11-year-old girl who had suffered long-term, intermittent chest pain, cough, fever, pleural effusions, and large pericardial effusions (PEs).

## Case Presentation

**Figure 1:**
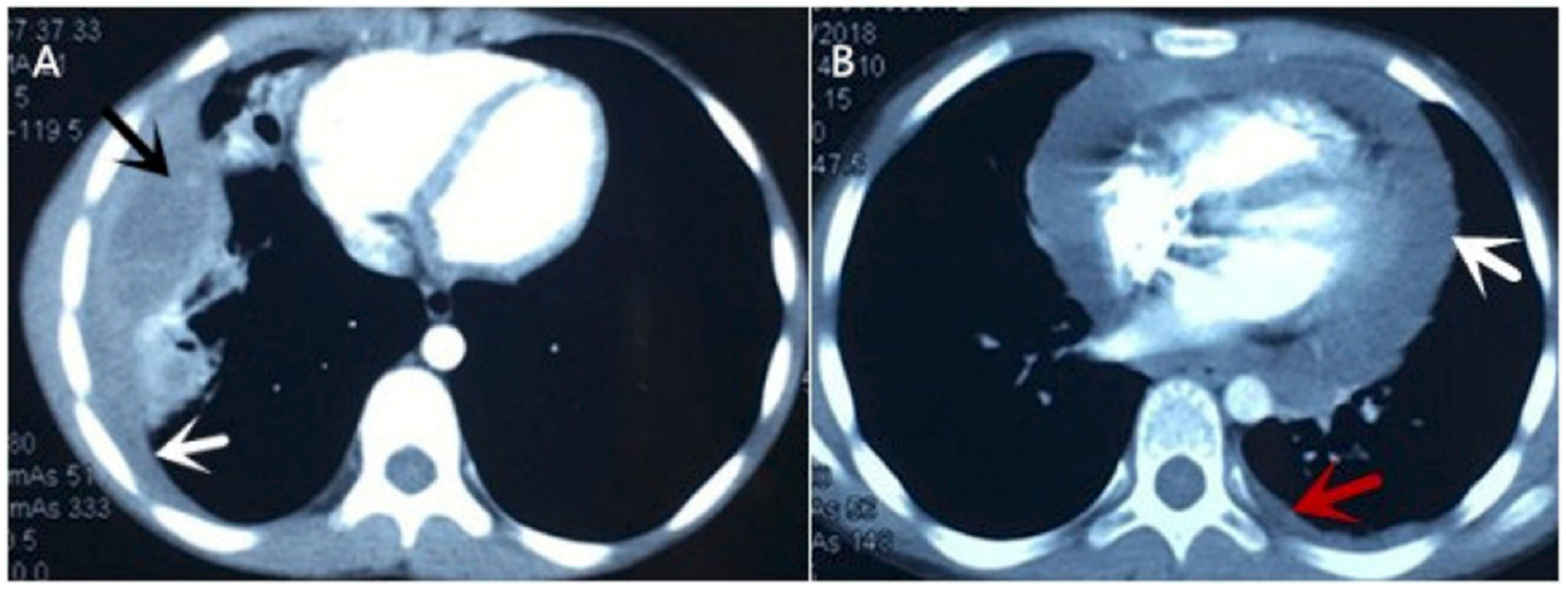
Chest CT of current cases. **(A)** It showed abscess in the middle and lower lobes of the right lung (black arrow), thickening of adjacent pleura, small amount of pleural effusion (white arrow). **(B)** It revealed large pericardial effusion (white arrow) and a small amount of pleural effusion on the left (red arrow).

An 11-year-old girl was admitted to our hospital in July 2019, when the recurrent chest pain, cough, and fever she had been experiencing for 13 months worsened. At first, the patient was admitted to a local hospital with chest pain, a cough and fever for 8 days in June 2018. Routine blood tests indicated leukocytosis of 15.69 × 10^9^/L, high eosinophil of up to 14.3%, a high platelet count of 456 × 10^9^/L, and raised C-reaction protein levels of up to 150.6 mg/L. Tests for tuberculin pure protein derivative was negative. A chest CT showed an abscess in the middle and lower lobes of the right lung, thickening of adjacent pleura, a small amount of pleural effusion, enlargement of the right hilar, and mediastinal lymph nodes (Figure 1A). A chest ultrasonography revealed pleural effusion 0.7 cm wide in the right pleural cavity, a 3.0 ×1.5 cm hypoechoic mass in the lower lobe of the right lung, and pericardial effusion. The patient received a right thoracotomy exploration, which showed pleural adhesions cauterization, right thoracic abscess clearance, and intercostal nerve block closure. Antibiotics ceased to be effective after 4 months of treatment. Pulmonary tuberculosis was considered, and anti-tuberculosis treatment (isoniazid and rifampicin) was administered for 4 months after admission. The patient was discharged 2 weeks later and was given an anti-tuberculosis drug for the following 6 months.

**Figure 2:**
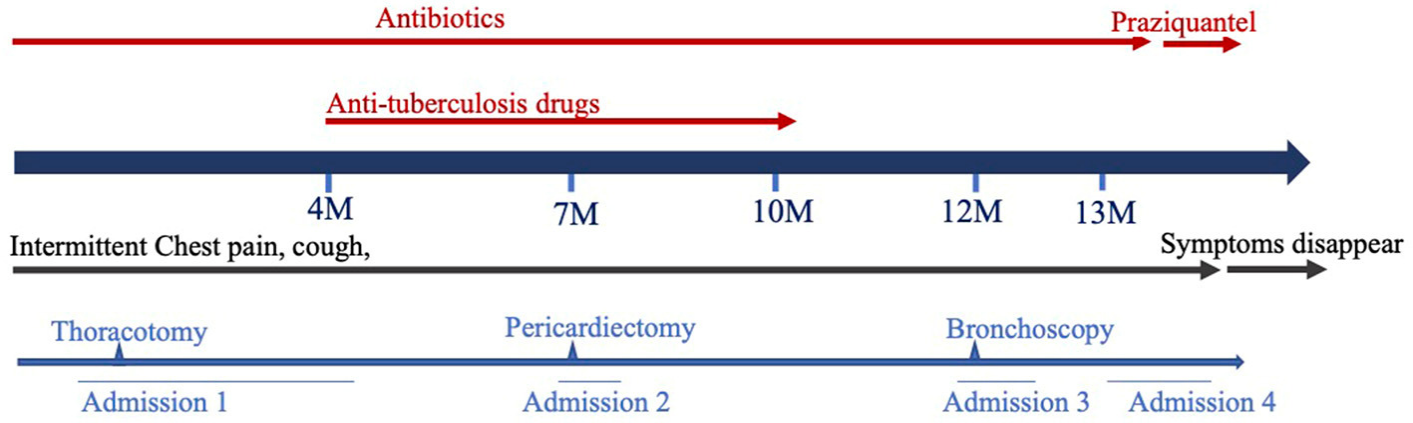
Diagrammatic representation of the symptoms and treatments.

Later, the patient was admitted to another hospital, as she was still suffering from chest pain, a cough and a fever 2 months after she was originally discharged. Her symptoms and laboratory tests were similar to those of described above, except that an echocardiography revealed that she had a large pericardial effusion (2.4 cm depth of the maximal diastolic separation) with increased echogenicity within fluid, which suggested exudative effusion. A chest CT revealed large PEs and light pleural effusion on the left (Figure 1B). Furthermore, tests for a tuberculosis infection were all negative. A thoracoscopy and partial pericardiectomy were performed, and a pericardium biopsy revealed chronic suppurative inflammation with granulation tissue hyperplasia. The echocardiography showed that PE had disappeared after the pericardiectomy. Antibiotics and anti-tuberculosis treatment were administered continuously. The patient was discharged because her symptoms abated after 1 month from her admission to the hospital (Figure 2). After discharge, she experienced intermittent chest pain accompanied by a frequent fever and a cough but was not hospitalized for 5 months. She was re-admitted to the hospital, and a bronchoscopy was performed without significant improvement. Following this, the patient was transferred to our hospital for further diagnosis and treatment.

She had no history of ingesting freshwater crabs or crayfish, but was born in Yunnan province where paragonimiasis is a severe public health issue. Her heart and lungs auscultation were normal. Her liver and spleen were also impalpable. No enlarged lymph nodes or obvious subcutaneous nodules were found in her whole body. After admission, a white blood cell count of 11.56 × 10^9^/L and eosinophils of up to 17% were noted. All tuberculosis-related tests (PPD test, T-spot, sputum smear for acidophilus and DNA of *Mycobacterium tuberculosis*) were negative. Renal and hepatic function tests were normal. A chest ultrasonography revealed pleural effusion of 0.5 mm depth in the left lung. No pericardial effusion was noted by a chest CT; however, ELISA for *Paragonimus* was positive, which confirmed the diagnosis of paragonimiasis. She was given 600 mg of praziquantel 3 times a day. After treatment, pleural effusion was reduced and her symptoms improved. The patient was discharged after 3 days of praziquantel therapy. The patient received another course of praziquantel therapy in the outpatient clinic 1 week later. The patient's chest pain accompanied by a fever and a cough did not recur.

## Discussion

*Paragonimus* genus has a complex life cycle that includes snails and crustaceans as its intermediate hosts, and mammals as its definitive hosts (6). It requires 2 intermediate hosts in its parasitic cycle. The first host is a mollusc (river snail), where the embryonated eggs become cercariae; and the second is a freshwater crustacean (crayfish), where they evolve to metacercariae. They pass to the definitive host (human or carnivorous mammal) when they are ingested (2, 4). Afterwards, the metacercariae enter the digestive tract, shell, then pass as larvae through the intestinal wall. Finally, the larvae enter the abdominal cavity and can even penetrate the diaphragm into the lung. During migration, the larvae and metabolites can cause severe symptoms as a result of mechanical damage, host damage, and allergic reactions. Paragonimiasis is a severe public health issue in south-west China. A previous investigation showed that Sichuan, Yunnan, Chongqing and Guizhou provinces in China are high-incidence areas of paragonimiasis. The water level of the Yangtze River rose significantly after the construction of Three Gorges Dam, resulting in more streams that are suitable for crab reproduction (7). The situation of our patient, who lives in an area where the parasite is endemic and always drank water that had not been boiled first, displayed all the conditions described above. Therefore, knowing the endemic context is essential in the diagnosis of paragonimiasis.

The symptoms of pulmonary paragonimiasis are non-specific and include a fever, chest pain, a chronic cough with rusty-brown sputum, and even hemoptysis (8). These are often confused with those of tuberculosis and lung carcinomas. In addition, paragonimiasis may also directly damage the liver and brain, and especially cause pericardial effusion, although hepatomegaly and splenomegaly may also be induced by hydropericardium (9). In the case of this patient, it is possible that the immune response induced by paragonimiasis was the main cause of exudative pericardial effusion. However, the large PEs noted in our patient are rarely reported to our knowledge. The etiology of PEs in children is complex. Infectious factors are the most important, among which suppurative, tuberculosis, and paragonimiasis are the 3 main causes. Therefore, when the patient has long-term chronic pericardial effusion accompanied by a fever, cough, and pleural effusion, parasites should also be considered.

It is difficult to distinguish paragonimiasis from tuberculosis because there are some similar clinical presentations. Our patient was misdiagnosed with pulmonary tuberculosis for over 6 months, which delayed the timely beginning of proper treatment. Hence, paragonimiasis should be considered in the differential diagnosis of pulmonary tuberculosis, especially in patients with a poor response to anti-tuberculotic chemotherapy (e.g., those that still have chest pain, a fever or a cough). The location of the patient in certain cities may be important when distinguishing paragonimiasis from tuberculosis, as well as their living habits, epidemiological history, and abnormal laboratory results. As with our patient, the facts of living in the areas where paragonimiasis is endemic and always drinking water that is not boiled first may be provided to diagnose paragonimiasis. Moreover, paragonimiasis causes eosinophilia, which is common in allergic diseases, parasitic infections, eosinophilic gastritis, hypereosinophilic syndrome, and mycotic infection, but not in tuberculosis (10). Chest CTs can play an important role in the differential diagnosis of paragonimiasis and tuberculosis. Clinicians should correctly interpret the CT imaging changes of paragonimiasis, such as “tunnel sign” and “migratory disease”, and should pay more attention to analyzing the differences between chest CTs and films showing the different stages of paragonimiasis and tuberculosis. It has been reported that the sensitivity and specificity of ELISA for *Paragonimus* species were 90.2 and 100.0%, respectively (11). The test should be performed as soon as possible to provide a rapid diagnosis, as the likelihood of finding the eggs of *Paragonimus* in a patient's feces and sputum is low (12).

In conclusion, in children with long-term chest pain, a cough, pleura, and PEs, especially those with a history of consuming raw food or drink, having eosinophilia, and not responding to treatment for pulmonary tuberculosis, paragonimiasis should be considered in the differential diagnosis. Early pathogen detection should be performed.

## Data Availability Statement

The original contributions presented in the study are included in the article/supplementary material, further inquiries can be directed to the corresponding author/s.

## Author Contributions

L-FT conceptualized and designed the study. JC drafted the initial manuscript. LQ collected the data and followed up the prognosis. All authors have read and agreed to the published version of the manuscript. All authors contributed to the article and approved the submitted version.

## Funding

This research was supported by the National Natural Science Foundation (81470214 and 82070028).

## Conflict of Interest

The authors declare that the research was conducted in the absence of any commercial or financial relationships that could be construed as a potential conflict of interest.

## Publisher's Note

All claims expressed in this article are solely those of the authors and do not necessarily represent those of their affiliated organizations, or those of the publisher, the editors and the reviewers. Any product that may be evaluated in this article, or claim that may be made by its manufacturer, is not guaranteed or endorsed by the publisher.
